# Intertrochanteric fracture non-unions with implant failure of the gamma nail

**DOI:** 10.11604/pamj.2016.23.57.7965

**Published:** 2016-02-29

**Authors:** Mouhcine Sbiyaa, Adil El Alaoui, Mohammed Admi, Kamal Lahrach, Amine Marzouki, Fawzi Boutayeb

**Affiliations:** 1 Service de Chirurgie Orthopédique et Traumatologique (A), Pr F. Boutayeb, CHU Hassan II de Fès, Maroc

**Keywords:** intertrochanteric fracture, complication, fracture non union

## Abstract

Failure of internal fixation of intertrochanteric fracture is associated with delayed union or malunion resulting in persistent pain and diminished function. We report a rare case of implant failure of the gamma nail with intertrochanteric fracture non union treated by DCS plate screw and bone graft.

## Introduction

Intertrochanteric fractures account for 10-34% of all hip fractures [1, 2]. The incidence of pertrochanteric femoral shaft fractures has a bimodal age distribution, affecting young patients following high-energy trauma (resulting in significant fracture comminution) and older patients after low velocity trauma secondary toosteoporosis or metastatic pathological lesions [3, 4]. The gamma interlocking nail was designed especially for the treatment of unstable peri and subtrochanteric femoral fractures [5, 6]. It combines the advantage of the sliding hip screw, a locked intramedullary implant with a lower bending moment, and increased length- and rotational stability [7, 8]. Because of its material strength, design, and mechanical advantage [9, 10] implant failure of the Gamma nail has been thought to be rare. We present a rare case of implant failure of the Gamma nail.

## Patient and observation

**Figure 1:**
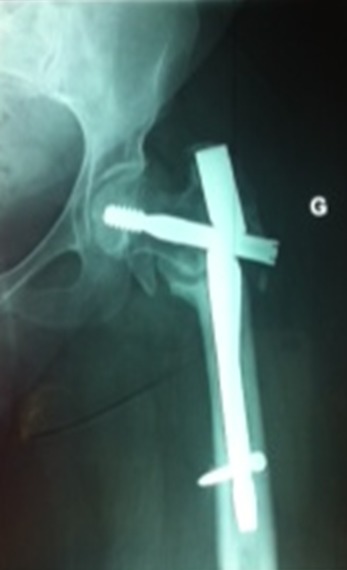
Antero-posterior radiograph of the left hip 6 months after stabilisation of peritrochanteric fracture with a gamma nail

**Figure 2:**
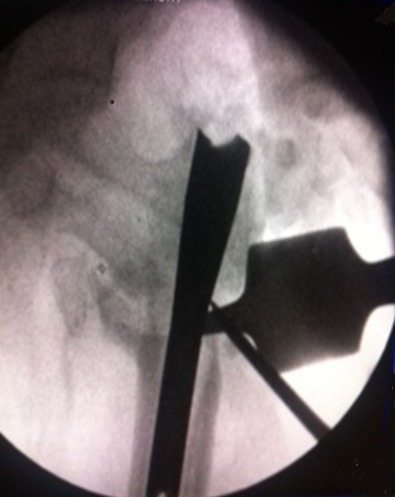
Intra-operative antero-posterior radiograph of materiel ablation

**Figure 3:**
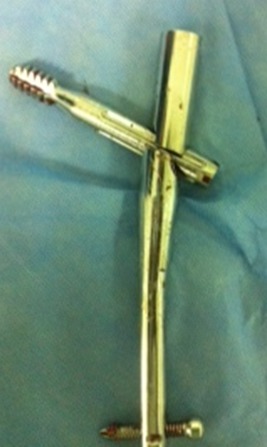
Fracture of gamma nail

**Figure 4:**
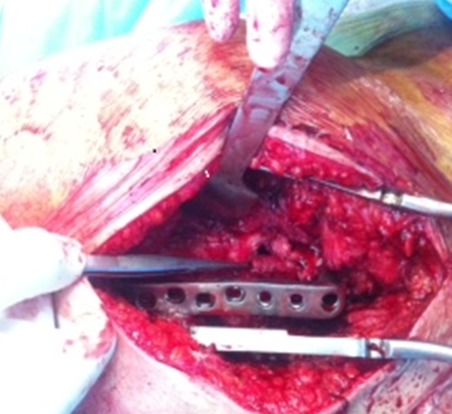
Intra-operative picture illustrating stabilisation of the non-union

**Figure 5:**
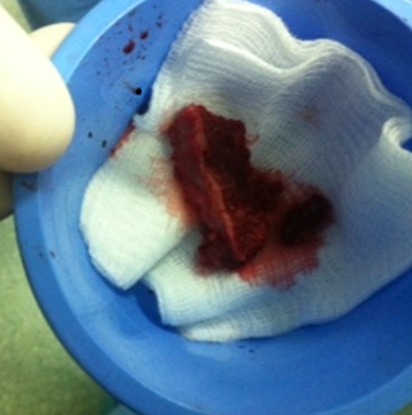
Bone graft levied from the iliac crest

**Figure 6:**
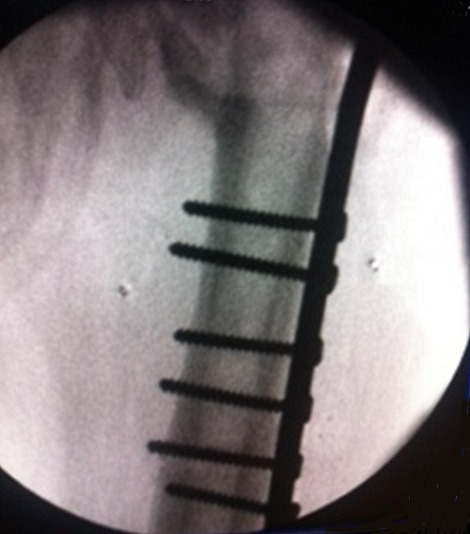
Intra-operative antero-posterior radiograph of the revision fixation

**Figure 7:**
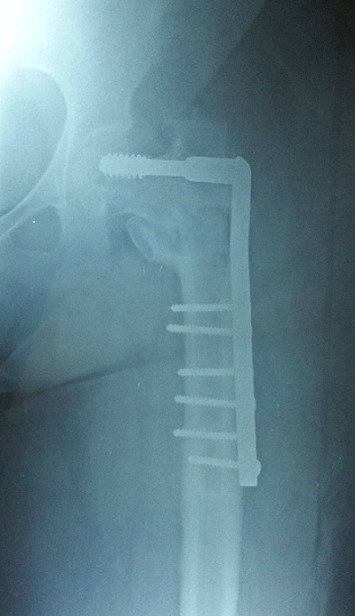
Antero-posterior radiograph of the left proximal femur showing the union of the fracture

The patient was a 67-year-old female with a good life quality, who presented an unstable intertrochanteric fracture of his left femur, Kyle and Gustilo type III following a fall at home. She underwent surgical fixation of his fracture with a short trochanteric Gamma nail, with a cervico diaphyseal angle of 130° and a distal diameter of 11 mm. The cephalic screw was 90 mm long; proximal static locking was performed. Postoperative radiographs showed acceptable reduction of the fracture. Four months later, the patient started complaining of permanent pain in his left hip, which became more severe over a few days, resulting in complete disability. He did not recall any trauma or unusual efforts. On physical examination, there was pain on palpation of the left trochanter as well as on mobilisation, particularly in rotation. The surgical wound was unremarkable. Radiographs showed breakage of the nail, at the opening for the cervical screw (Figure 1), resulting in an angulation between the nail and the cephalic screw. The fracture showed no signs of healing; the fracture line was still visible, with sclerosis ofthe bone ends, typical for a nonunion. The broken nail was removed (Figure 2, Figure 3) and a DCS plate screw was implanted with bone graft levied from the iliac crest (Figure 4,Figure 5, Figure 6). Early weight-bearing was encouraged. All bacteriological samples taken were sterile. Two months later, radiographs showed healing of the fracture (Figure 7). The patient is presently asymptomatic, walking without help.

## Discussion

Unstable peri and subtrochanteric fractures of the proximal femur are complicated by the massive tension moments laterally and compressive forces medially created by the weight of the body, hip flexors and external rotators and by the abductor musculature, resulting often in fracture displacement, loss of fixationand implant failure [11, 12]. The gamma nail proved tobe an adequate implant to stabilize stable and unstableperi- and subtrochanteric fractures. In the recent literature, the incidence of intraoperative fracture of the shaft was reported to be decreased because of the modification of implant design and the improvement of surgical technique [13–16]. The reported incidence of implant failure of the Gamma nail is 0%-0.4% in multicenter studies [17–19]. In a series of 2500 Gamma nail fixations, only 4(0.16%) nails broke, all associated with nonunion and continued weight bearing. Breakage time varied from postoperative month 6 to 15, suggesting fatigue caused by dynamic loading [19]. A weak point in the Gamma nail seems to bearound the opening for the cephalic screw, where the cross section narrows, approximately by 73% [20]. This is the critical zone where forces coming from the femoral neck are transmitted to the diaphyseal nail [19, 21]. If the guide for the cervical screw is not properly placed, inappropriate drilling of the nail or off-centre introduction of the cervical screw may cause erosion of the nail in the cervical opening. This complication usually occurs late, 6 to 10 months after surgery. Thus, the possibility of the implant being broken when there is recurring pain at the operated hip or even more frequently at the thigh must be taken into consideration. Specialattention must be paid to those cases with pathological fractures [22]. We recommend taking radiographs of the operated hip in two different projections in the follow-ups and the option of dynamisation of the device and/or bone graftingmust always be considered when delayed union is suspected. The options for treatment will depend on each particular situation. Retrieving the implant may bevery difficult, especially the distal fragment of the broken nail. It may be necessary to open a window in the diaphyseal cortex. For this reason, we recommend a careful preoperative planning and rigorous technique that will avoid problems with the screws, both proximal and distal.

## Conclusion

Breakage of the Gamma nail due to fatigue is avery rare complication. It occurs 6 to 10 months after surgery. It is a consequence of nonunion at the fracture site. The weakest point of the Gamma nail is theopening for the cervical screw.The best therapeutic option will depend on each particular situation.
